# Brainana: an end-to-end preprocessing framework for macaque neuroimaging

**DOI:** 10.64898/2026.06.03.729972

**Published:** 2026-06-08

**Authors:** Xingyu Liu, Yijun Zhang, Zi Yin, Zonglei Zhen, Michael J. Arcaro

## Abstract

Macaque MRI bridges non-invasive systems neuroscience with cellular and circuit-level mechanisms, but preprocessing remains fragmented across tools that are difficult to integrate, adapt to non-human primate acquisitions, and deploy reproducibly. We present Brainana, an automated, BIDS-compatible preprocessing framework for macaque neuroimaging. Brainana integrates structural and functional preprocessing, cortical surface reconstruction, quality control, transform tracking, and atlas projection within a containerized package, with cloud access for users without local compute. It incorporates macaque-trained deep learning models for brain extraction and tissue segmentation, conformation to standardize variable acquisitions, and surface reconstruction optimizations for macaque neuroanatomy. Across 23 imaging sites, Brainana processed data spanning heterogeneous scanners, protocols, species, and resolutions, yielding accurate anatomical correspondence across 130 monkeys, reliable native-space cortical surfaces, localized task-evoked activations, and reproducible brain-wide resting-state correlation structure. Brainana enables reproducible, scalable, and accessible macaque MRI preprocessing that supports cross-study comparison and multimodal integration across spatial scales, from neurons to networks.

## Full Text Availability

The license terms selected by the author(s) for this preprint version do not permit archiving in PMC. The full text is available from the preprint server.

